# Pharmacological inhibition of frizzled 4 delays cell cycle progression and limits oral squamous cell carcinoma growth

**DOI:** 10.3389/fcell.2026.1756565

**Published:** 2026-02-17

**Authors:** Riccardo Destefani, Ilaria J. Valookkaran, Sabrina Bensland, Christian T. Meisel, Cristina Porcheri

**Affiliations:** Institute of Oral Biology, Faculty of Medicine, University of Zurich, Zurich, Switzerland

**Keywords:** FZD4, FzM1, oral squamous cell carcinoma, retinoic acid, Wnt pathway

## Abstract

Oral squamous cell carcinoma is an aggressive malignancy driven by aberrant signaling pathways, with Wnt signaling acting as a central regulator of proliferation, tumor-microenvironment interactions, and oncogenic growth. Here, we focus on Frizzled-4, a Wnt receptor with context-dependent functions in epithelial cancers. We show that pharmacological inhibition of Frizzled-4 delays cell cycle progression, and reduces proliferative capacity. These effects are accompanied by suppressed metabolism of retinoic acid, suggesting that Frizzled-4 inhibition stabilizes intracellular retinoic acid levels, which ultimately contributes to the observed cell cycle slowdown. Blocking retinoic acid signaling reverses the cell cycle effects of Frizzled-4 inhibition, confirming that retinoic acid-dependent regulation operates downstream of Frizzled-4. By restraining uncontrolled proliferation and attenuating oncogenic signaling, Frizzled-4 emerges as a key molecular node in oral squamous cell carcinoma, highlighting its potential as a therapeutic target to counteract Wnt-driven tumor growth.

## 1 Introduction

Oral squamous cell carcinoma (OSCC) is the most prevalent oral malignancy, accounting for over 90% of cancers of the oral cavity. It remains associated with poor prognosis and high mortality despite advances in diagnosis and treatment (Johnson et al., 2020; Ferlay et al., 2019; Bray et al., 2018). Traditionally linked to tobacco use, alcohol consumption, and human papillomavirus (HPV) infection, OSCC is now recognized as a multistep disease driven by the dysregulation of interconnected signaling networks governing proliferation, survival, and tumor-microenvironment interactions (Cramer et al., 2019). At the molecular level, OSCC progression reflects the convergence of oncogene activation, tumor suppressor loss, epigenetic alterations, and aberrant receptor-mediated signaling. Dysregulation of proliferative pathways, particularly the epidermal growth factor receptor (EGFR)/mitogen-activated protein kinase (MAPK) cascade, amplifies growth and survival signals, promotes metabolic reprogramming, and contributes to therapeutic resistance (Horn et al., 2015; Cheng et al., 2022), while crosstalk with inflammatory mediators such as Nuclear Factor kappa B (NF-κB) and altered apoptotic regulators further drives tumor progression (Li et al., 2019). Frequent mutations in tumor suppressors, most notably TP53, compromise DNA damage checkpoints and genomic stability, while disruption of the retinoblastoma (Rb) pathway through cyclin-dependent kinase (CDKs) hyperactivation leads to inappropriate release of Early 2 Factor (E2F) transcription factors, driving premature S-phase entry and uncontrolled cell-cycle progression (Johnson et al., 2020; Stransky et al., 2011; Sequeira et al., 2020; Yoo et al., 1994; Pande et al., 1998; Nakahara et al., 2000). Emerging evidence highlights epigenetic mechanisms and non-coding RNAs as critical modulators of oncogenic signaling, collectively establishing a permissive environment for sustained proliferation and impaired growth control (Kalantary-Charvadeh et al., 2024; Kabzinski et al., 2021; Kumar et al., 2024).

Notably, many of these regulatory mechanisms converge on the Wingless and Int-1 (Wnt) signaling network, a central hub for cellular proliferation, differentiation, and homeostatic control (Yuan et al., 2019). Dysregulated Wnt activity, whether through direct mutations, aberrant expression or secondary modulation, has emerged as a critical driver of tumor initiation and maintenance in OSCC (Ishida et al., 2007; Laxmidevi et al., 2010; Moles et al., 2016; Xie et al., 2020).

Wnt signaling is a highly conserved molecular cascade that regulates fundamental cellular processes including fate determination, proliferation, migration, and polarity during embryonic development and tissue homeostasis (Clevers and Nusse, 2012; Logan and Nusse, 2004). In the canonical Wnt pathway, Wnt ligands bind to Frizzled (FZD) receptors and co-receptors low-density lipoprotein receptor-related proteins 5 and 6 (LRP5/6), leading to inhibition of the β-catenin destruction complex. Stabilized β-catenin accumulates in the cytoplasm and subsequently translocates into the nucleus, where it interacts with transcription factors to activate target genes controlling growth and differentiation. In mammals, ten distinct FZD receptors have been identified, each contributing to Wnt-mediated regulation in a tissue-specific manner. Among them, Frizzled-4 (FZD4) regulates vascular integrity, neural development, and tissue homeostasis through β-catenin–dependent signaling. Its activity ensures controlled proliferation and differentiation, whereas its dysregulation leads to developmental disorders and tumorigenesis in a context-dependent manner (Yang S. et al., 2018; Sompel et al., 2021). In non-small cell lung carcinoma, colon cancer, prostate cancer, and hematologic malignancies, FZD4 has been characterized as a pro-oncogenic driver, enhancing epithelial-to-mesenchymal transition (EMT) and promoting cell survival through activation of the canonical Wnt/β-catenin pathway (Freeman, 2008; Formosa et al., 2014; Pu et al., 2013; Bassett et al., 2016; Tickenbrock et al., 2008; Gupta et al., 2010). Conversely, in cutaneous squamous cell carcinoma, FZD4 expression is downregulated, and experimental overexpression inhibits proliferation and induces apoptosis, suggesting a tumor suppressor function in this context (Zhang et al., 2021).

FZD4 is frequently overexpressed in OSCC, although its specific mechanistic role in tumor development and progression remains to be fully elucidated (Lu and Xie, 2023). To explore the therapeutic potential of this receptor as a pharmacological target, we investigated the effects of FZD4 inhibition in OSCC. Using next-generation sequencing assays combined with molecular and kinetic analyses, we found that blocking FZD4 delayed cellular proliferation through a retinoic acid (RA)-dependent regulation. Together, our findings establish FZD4 as a clinically relevant target in OSCC and highlight the central role of the Wnt-retinoic acid axis in regulating malignant tumor growth.

## 2 Materials and methods

### 2.1 Animal work

C57Bl/6 mice were handled according to Swiss Animal Welfare regulations. The study was approved by the Cantonal Veterinary Office in Zurich (licenses ZH197/2017 and ZH086/2021). To induce OSCC, mice were given drinking water containing 50 μg/mL 4-nitroquinoline 1-oxide (4NQO; #N8141, Sigma-Aldrich) from 6 to 22 weeks of age. Control mice received water with the same amount of Dimethyl sulfoxide (DMSO) (#D4540, Sigma-Aldrich). After 16 weeks of treatment, mice continued on normal water until euthanasia (32 weeks).

### 2.2 Immunostaining

After tissue collection, samples were fixed in 4% paraformaldehyde (PFA) for 1 h at 4 °C, then cryoprotected in 30% sucrose. The tissues were embedded in cryo-embedding medium (Tissue-Tek O.C.T. Compound, Sakura) and stored at −80 °C until further processing. Sections of 10 µm thickness were cut using a cryostat (Leica CM3050S) and kept at −80 °C until staining.

For immunofluorescence analysis, cryosections were first permeabilized with 0.5% Triton X-100 in Phosphate-buffered saline (PBS) for 1 h at room temperature, followed by blocking in a solution containing 10% fetal bovine serum (FBS) and 0.1% Triton X-100. Sections were then incubated overnight at 4 °C with the primary antibody anti-FZD4 (#SAB2108140, Sigma-Aldrich; 1:200) diluted in blocking buffer. After washing with PBS, sections were incubated with the secondary antibody donkey anti-rabbit IgG Alexa Fluor 488 (1:1000; Invitrogen). Nuclei were counterstained with DAPI (4′,6-diamidino-2-phenylindole dihydrochloride; #D1306, Invitrogen). Finally, slides were mounted using Mowiol 4–88 (#81381 and #G-6279, Sigma-Aldrich). Fluorescent images were acquired using a Leica DM6000B fluorescence microscope.

### 2.3 Cell culture

The SCC-25 epithelial cell line, originating from the tongue of a 70-year-old OSCC patient, was obtained from ATCC/LGC (American Type Culture Collection, SCC-25CRL-1628™). SCC25 were maintained in Dulbecco’s Modified Eagle Medium: Nutrient Mixture F-12 (DMEM/F12) medium supplemented with 1.2 g/L sodium bicarbonate, 2.5 mM L-glutamine, 15 mM 4-(2-hydroxyethyl)-1-piperazineethanesulfonic acid (HEPES), 0.5 mM sodium pyruvate (#11330032, Gibco-ThermoFisher), and 10% FBS (#16000044, Gibco-ThermoFisher). Once cultures reached ∼80% confluence, cells were passaged using trypsin/Ethylenediaminetetraacetic acid (EDTA) (#15400054, Gibco-ThermoFisher) and cryopreserved at a density of 10^6^ cells/vial in 10% DMSO (#D4540, Sigma-Aldrich). For experimental treatments, low passage (<20 passages) SCC-25 cells were exposed to 3-Hydroxy-5-[[(2-Naphthalenylamino)carbonyl]amino]benzoic acid (FzM1) (10µM; # HY-116553, MedChemExp), FzM1+ Retinoic acid receptor inhibitor (RARi) (AGN 193109 100nM; #HY-U00449, MedChemExp) or vehicle control DMSO (#D8418, Sigma-Aldrich) for 5 days.

### 2.4 Cell immunofluorescence

SCC-25 cells were seeded at a density of 20,000 cells per well in a 12-well plate with coverslips (Electron Microscopy Sciences #72196). Fixation was carried out with 4% PFA or cold 100% MetOH for 15 min followed by PBS washes. Cells were then permeabilized for 30 min with 0.5% Triton and subsequently blocked in PBS containing 10% FBS and 0.1% Triton for 1h RT. Primary antibodies were diluted in blocking solution and incubated overnight. For immunofluorescence, cells were probed with anti-FZD4 (#SAB2108140; Sigma-Aldrich, 1:100), anti-phospho Histone3 (pH3) (#9701, Cell Signaling, 1:300); anti-Ki67 (#ab16667, abcam, 1:100) and detected with donkey anti-rabbit IgG Alexa Fluor 488 or 546 (1:1000; Invitrogen). Nuclear staining was performed using DAPI (4′,6-diamidino-2-phenylindole, dihydrochloride; #D1306, Invitrogen). Samples were mounted using Mowiol 4–88 and glycerol (#81381 and #G-6279, Sigma-Aldrich). Immunofluorescence images were produced using a Leica DM6000B microscope. Quantifications were performed in five different fields of view for each sample (n = 3).

### 2.5 Kinetic study

To calculate the growth fraction upon treatment, SCC25 cells were plated on coverslips and treated with DMSO (control), FzM1 (treated) or FzM1+RARi (treated) for 5 days before kinetic analyses. Cells were exposed to 10 μM 5-Ethynyl-2′-deoxyuridine (EdU, MedChemExp, # HY-118411) for 1h, 4h, 8h, 10h, 12h, 14h, 16h, 20h, 24h, 38h, and 60 h. After incubation time, cells were fixed in 4% PFA and stained for EdU using the VF 488 Click-iT EdU Universal Cell Proliferation Detection Kit (MedChemExp). Nuclear staining was performed using DAPI (4′,6-diamidino-2-phenylindole, dihydrochloride; #D1306, Invitrogen). Samples were mounted using Mowiol 4–88 and glycerol (#81381 and #G-6279, Sigma-Aldrich). Immunofluorescence images were produced using a Leica DM6000B microscope. Quantifications were performed in five different fields of view for each sample (n = 3). Calculation of cell cycle length (Tc) and S-phase length (Ts) were extrapolated from the function obtained graphically of the growing phase (Nowakowski et al., 1989). Quantification was carried out using ImageJ and FIJI software (Schindelin et al., 2012; Schneider et al., 2012). Graphs and statistical analyses were performed using Prism software (GraphPad, GraphPad Software, San Diego California USA; http://www.graphpad.com/). All data were analyzed using the two-tailed Student's t-test. P < 0.05 were considered statistically significant.

### 2.6 Western blot

Treated SCC-25 cells were harvested in Radioimmunoprecipitation assay (RIPA) buffer (#9806; Cell Signaling) supplemented with protease inhibitors (#11836170001; Roche) and homogenized following scraping. Proteins were precipitated by adding cold acetone to the lysates and incubating for 1 h at −20 °C. The samples were then centrifuged at 15,000 rpm for 10 min, and the resulting protein pellets were resuspended in a buffer mixture containing RIPA buffer, Laemmli Buffer (LB, #1610747, Bio-Rad), and dithiothreitol (DTT, #7016, Cell Signaling). For Sodium Dodecyl Sulfate-PolyAcrylamide Gel Electrophoresis (SDS-PAGE), 20 ug of protein lysate was loaded per lane and separated on 6%–10% Mini-PROTEAN TGX Gels using the Mini-PROTEAN system (#1658005, Bio-Rad) under 70 V constant voltage. Protein transfer was carried out with the Trans-Blot Turbo system (#1704150, Bio-Rad) using Trans-Blot Turbo Mini 0.2 µm Nitrocellulose Transfer Packs (#1704158, Bio-Rad), following the standard Turbo protocol for 30 min. Blocking of the membrane was performed in 5% non-fat dry milk in Tris-Buffered Saline (TBS) or 1% Bovine Serum Albumin (BSA) in TBS for 1h at room temperature. Primary antibody used were incubated overnight at 4 °C in a shaking condition. Antibodies used were: a- glyceraldehyde-3-phosphate dehydrogenase (GAPDH)- Horseradish Peroxidase (HRP) conjugated (Clone YA874, MEC, #HY-P80951A; 1:1000); a-Keratin10 (Krt10) (Covance, 1:1000). Membrane was washed in TBS-Tween® 20 (TBS-T, Tween20 #P9416; Sigma-Aldrich) and TBS for 1h at RT in shaking conditions. Species-specific secondary antibodies a-rb HRP conjugated (Abcam, #ab97051, 1:1000) were used.

### 2.7 RNA isolation

Treated SCC-25 cells were pelleted and snap-frozen in liquid nitrogen prior to RNA isolation following the manufacturer’s protocol (RNeasy Mini Kit; Qiagen). Genomic DNA was removed via gDNA removal columns and further purified. Total RNA yield was quantified using a NanoDrop spectrophotometer (Thermo Fisher Scientific, Waltham, USA). For cDNA synthesis, 500 ng of RNA was reverse transcribed with the iScript™ cDNA Synthesis Kit (Bio-Rad Laboratories AG, Cressier FR, Switzerland) according to the supplier’s instructions.

### 2.8 Quantitative real-time PCR

Quantitative real-time PCR was conducted on the CFX Connect Real-Time System using CFX Manager software (Version 3.1). Gene expression analyses were carried out with SYBR® Green PCR Master Mix (Applied Biosystems, Carlsbad, CA, USA) together with the corresponding oligonucleotide primers (Supplementary Table S1). Exon-exon primers were designed using Primer3 software (Kõressaar et al., 2018). Hairpin, hetero-dimers and self-dimers formation was excluded (tollerance DeltaG >=-9 kcal/mol). Each reaction was run in technical duplicates across four biological replicates. Thermocycling conditions were as follows: initial denaturation at 95 °C for 10 min; 40 cycles of 95 °C for 15 s and 60 °C for 60 s. Melting curve analysis was performed under the following parameters: 95 °C for 15 s, 60 °C for 5 s, and 95 °C for 30 s. Relative expression levels were determined using the ΔΔCt method (2^−ΔΔCT^) after normalization to the Ct values of the housekeeping gene. Graphs and statistical analyses were performed using Prism10 software (GraphPad, GraphPad Software, San Diego California USA; http://www.graphpad.com/). All data were considered statistically significant when p < 0.05.

### 2.9 Transcriptomic analysis

Bulk RNA sequencing was performed as follows: Total RNA was isolated using the Qiagen RNeasy Mini Kit (Qiagen, #74104) with on-column genomic DNA elimination using RNase-Free DNase I (Qiagen, #79256), according to the manufacturer’s instructions. RNA quality was assessed prior to library preparation, and only samples with RNA integrity (RINe) scores >9.2 were used for downstream processing. High-quality RNA was reverse-transcribed into cDNA, followed by fragmentation and adapter ligation. Sequencing libraries were prepared using the Illumina TruSeq protocol and sequenced on an Illumina NovaSeq 6000 platform (200 million reads per sample). Raw sequencing data were quality-checked using FastQC and aligned to the reference genome with STAR. Gene expression levels were quantified by counting aligned reads with Kallisto and normalized as fragments per kilobase of transcript per million mapped reads (FPKM). Differentially Expressed Genes (DEG) were obtained from the statistical packages DEseq2 output (p.adj <= 0.01) (Supplementary Method 1). We determine pathway enrichment using the ReactomeGSA Bioconductor R package for pathway-based analysis (Johannes Griss, 2025) GSEA 4.0.3 (https://www.gsea-msigdb.org/gsea/index.jsp), Enrichr and Appyters (https://maayanlab.cloud/Enrichr/).

### 2.10 Data availability statement

The datasets generated for this study can be found in the BioProject PRJNA1355448 https://dataview.ncbi.nlm.nih.gov/object/PRJNA1355448?reviewer=3ct113p5bca2ami318gd1hpel9.

## 3 Results

### 3.1 FZD4 is expressed in murine and human OSCC models

**FIGURE 1:**
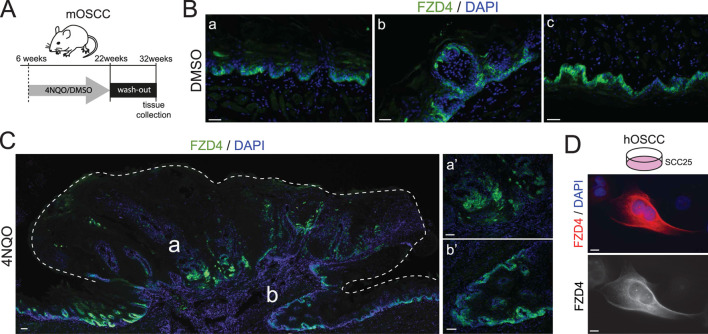
The WNT receptor FZD4 is expressed in murine and human models of OSCC. **(A)** Schematic representation of OSCC induction via 4NQO administration. Mice received 4NQO in drinking water for 16 weeks, followed by a 10-week washout period. **(B)** FZD4 expression in tissue cryosections from control mice. FZD4 immunofluorescence (green) is localized predominantly to the basal layer of the stratified epithelium in **(a)** the dorsal tongue, **(b)** fungiform papillae, and **(c)** the ventral tongue of DMSO-treated animals. Nuclei are counterstained with DAPI (blue). Scale bar, 30 µm. **(C)** FZD4 expression in tissue cryosections from 4NQO-treated mice. FZD4 immunoreactivity (green) is detected in clustered cells within the OSCC mass. Left panel: overview of carcinogenic lesion (dashed lines); right panels (a’, b’): higher magnifications of the corresponding areas in the overview. Nuclei are counterstained with DAPI (blue). Scale bars: overview, 100 μm; magnifications, 30 µm. **(D)** Human *in vitro* OSCC model showing positive FZD4 staining. Left panel: merged image of FZD4 (red) and nuclear DAPI counterstain (blue); right panel: single-channel FZD4 signal. Scale bar, 10 µm.

To elucidate the potential contribution of FZD4 to Wnt signaling activity in the context of OSCC, its expression was investigated *in vivo* using a 4NQO–induced murine model of OSCC (Sagheer et al., 2021). The carcinogen 4NQO was administered through the drinking water for 16 weeks, followed by a 10-week washout phase (Figure 1A). This protocol reliably induced malignant transformation with uniform penetrance, resulting in exophytic lesions on the dorsal surface of the tongue. In contrast, DMSO-treated control animals do not exhibit any local or systemic pathological manifestations. Following tissue collection, the tongues were embedded and cryosectioned for analysis. In control specimens, fluorescent immunolabelling revealed FZD4 expression restricted to the basal layer of the tongue epithelium (Figure 1B), with positive signal on both dorsal and ventral surfaces (Figures 1B,a,c). Positive staining was also detected in the fungiform papillae containing taste buds (Figures 1B,b). In 4NQO-treated mice, FZD4 immunoreactivity persisted within the dysplastic epithelium of the lesions, showing a clustered distribution pattern (Figure 1C, overview and magnifications).

To extend these findings to a human model, we analyzed SCC25 cells derived from the tongue of a 70-year-old male patient with squamous cell carcinoma (ATCC #CRL-1628) (Rheinwald and Beckett, 1981). Immunofluorescence confirmed FZD4 expression, displaying a membrane-associated, reticular pattern on the cell surface.

### 3.2 Blockade of FZD4 induces a distinct change in transcriptomic signature

To assess the functional relevance of FZD4 in OSCC, we utilized FzM1, a negative allosteric modulator of FZD4 specifically inhibiting the FZD4-WNT signaling axis in a non-competitive manner (Generoso et al., 2015). SCC25 cells were treated with DMSO (control group) or FzM1 (treated group), and total RNA was subsequently extracted for downstream analyses. A transcriptomic library was generated for bulk RNA sequencing, revealing 834 differentially expressed genes (Supplementary Method 1).

**FIGURE 2:**
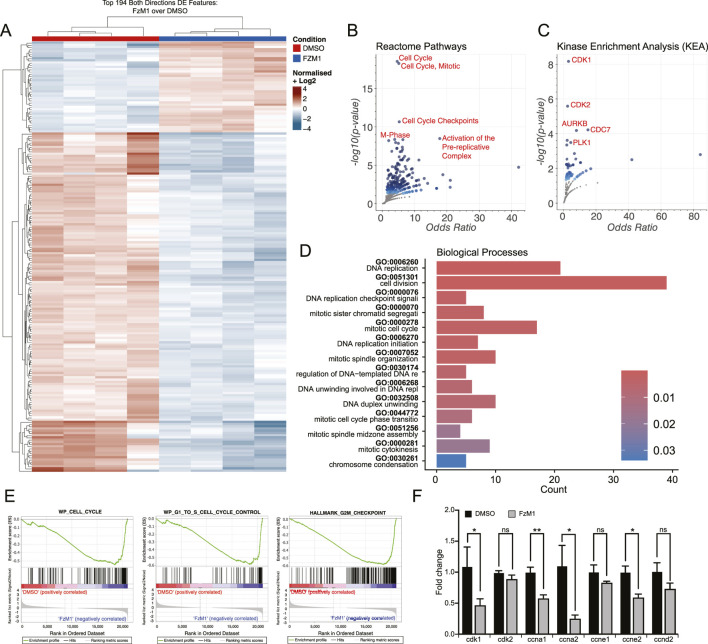
Transcriptomic Shifts in Cell Cycle Programs Following FZD4 Inhibition by FzM1. **(A)** Heatmap of differentially expressed genes (DEGs) between control DMSO- and FzM1-treated human SCC25 cells, generated by hierarchical clustering analysis. **(B,C)** Volcano plots of enriched terms from the GO Biological Processes gene set, obtained via Enrichr–Appyter analysis. Each point represents a single enriched term; larger and darker-colored points indicate higher statistical significance. Red labels highlight the top most significant GO terms based on p- and q-values. Left panel: Reactome Pathways gene set. Right panel: Kinase Enrichment Analysis gene set. **(D)** Bar chart illustrating over-representation analysis (ORA) results for upregulated GO Biological Process terms in FzM1-treated cells. **(E)** GSEA plots showing enrichment of pathways associated with cell cycle control across multiple gene sets. **(F)** Quantitative RT–PCR validation of selected transcripts showing altered expression following FzM1 treatment. Relative mRNA expression levels are shown as fold change (FC): *CDK1* (DMSO: 1.098 vs. FzM1: 0.47; p = 0.039), *CDK2* (DMSO: 1.0009 vs. FzM1: 0.8936; p = 0.167), *CCNA1* (DMSO: 1.0078 vs. FzM1: 0.58; p = 0.0037), *CCNA2* (DMSO: 1.1088 vs. FzM1: 0.256; p = 0.032), *CCNE1* (DMSO: 1.0108 vs. FzM1: 0.8363; p = 0.217), *CCNE2* (DMSO: 1.0079 vs. FzM1: 0.5982; p = 0.013), and *CCND2* (DMSO: 1.017 vs. FzM1: 0.73; p = 0.144). Data are presented as mean ± SEM. *p < 0.05; **p < 0.01; ns, not significant (*p* > 0.05).

The top 194 significant genes are visualized in the heatmap (Figure 2A). Pathway enrichment analysis using *Enrichr* Reactome Pathways and Kinase Enrichment Analysis (KEA) databases showed that most differentially expressed genes are associated with regulation of proliferation and cell cycle progression (Figures 2B,C). Gene Ontology (GO) analysis of biological processes further confirmed that the majority of affected categories relate to regulation of cellular kinetic, highlighting the transcriptional impact of FzM1 treatment on SCC25 proliferation (Figure 2D). Gene Set Enrichment Analysis (GSEA) consistently supported this observation, revealing altered expression of genes involved in the regulation of cell cycle progression (Figure 2E). To characterize these changes in greater detail, we analyzed the expression of individual transcripts by RT-PCR. Cyclin-dependent kinase 1 (CDK1) expression was markedly reduced upon FzM1 treatment, whereas other CDKs, such as Cyclin-dependent kinase 2 (CDK2), appeared mostly unchanged. Among the differentially expressed cyclins, FzM1 treatment led to decreased expression of cyclin A family members (A1 and A2) and cyclin E (E2) (Figure 2F).

### 3.3 Phenotypic analysis of proliferation reveals slower cell cycle in OSCC cells treated with FzM1

**FIGURE 3:**
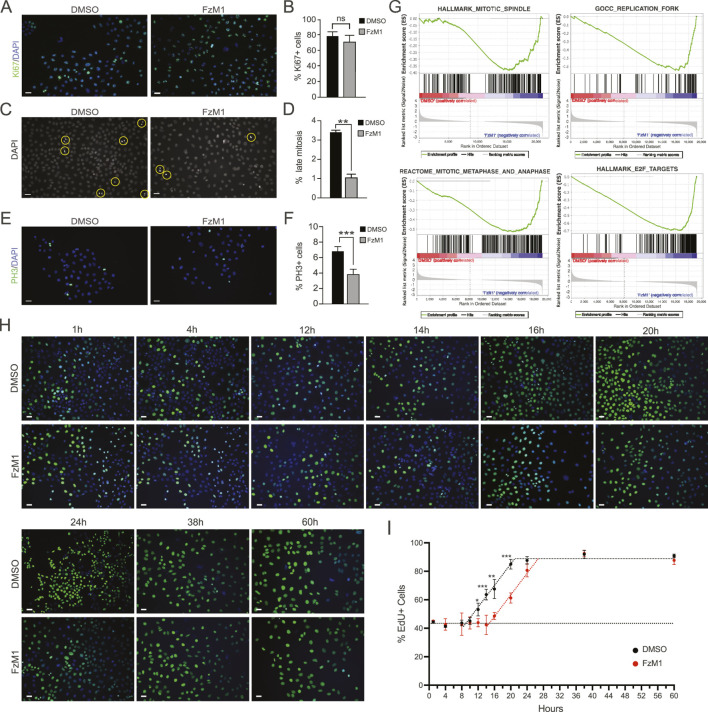
Phenotypic characterization of cell cycle alterations following FzM1 treatment. **(A)** Representative immunofluorescence images of Ki67 (green) with nuclear counterstaining using DAPI (blue). Scale bar, 30 µm. **(B)** Quantification of the percentage of Ki67-positive cells relative to total nuclei. **(C)** Mitotic figures identified morphologically via DAPI staining. Scale bar, 30 µm. **(D)** Quantification of the percentage of cells in mitosis relative to total nuclei. **(E)** Representative immunofluorescence images of phospho-histone H3 (PH3, green) with DAPI (blue) nuclear counterstaining. Scale bar, 30 µm. **(F)** Quantification of the percentage of PH3-positive cells relative to total nuclei. **(G)** GSEA plots showing enrichment of pathways associated with specific cell cycle phases, mitotic structural changes, and key cell cycle regulators. **(H)** Representative images of EdU-positive cells over time during cumulative incorporation assays used to calculate cell cycle progression. Scale bar, 30 µm. **(I)** Graphical representation and quantification of the percentage of EdU-positive cells over time for cell cycle analyses. p-values for significant time point: 14 h, p = 0.024; 16 h, p = 0.00045; 20 h, p = 0.00421; 24 h, p = 0.03402. *p < 0.05; **p < 0.01; ***p < 0.001; not significant (p > 0.05). Data are presented as mean ± SEM.

We then sought to phenotypically characterize the effects of FzM1 on proliferating OSCCs cells. Firstly, we investigated whether FzM1 induces cell cycle exit or quiescence by analyzing the expression of the proliferation marker Ki67. Since Ki67 is expressed during all active phases of the cell cycle (late G1, S, G2, and M) but absent in resting cells (G0), we quantified Ki67-positive cells following FzM1 treatment (Figures 3A,B). No significant changes were observed, indicating that FzM1 does not induce quiescence.

We then examined the cell cycle phase most affected by FzM1. Mitotic figures corresponding to metaphase, anaphase, and telophase were readily observed in both control and treated cells (Figure 3C). Quantification revealed a significant reduction in the number of cells undergoing active mitotic division upon FzM1 treatment (Figure 3D). Consistently, immunofluorescence for phospho-H3, a mitotic marker expressed from late G2 through advanced mitosis, showed fewer labeled cells in treated samples compared to controls (Figures 3E,F).

To further validate these observations, we performed GSEA to assess gene expression changes related to mitosis. FzM1 treatment was negatively correlated with gene sets associated with mitotic spindle assembly and metaphase-anaphase transition (Figure 3G left panels; Supplementary Figure S1). Additionally, GSEA identified differential expression of genes regulating S-phase progression and cell cycle progression mediated by E2F transcription factors (Figure 3G, right panels; Supplementary Figure S1). These findings suggest that cells are kept in cycle during FzM1 treatment, with several phases of the cycle being affected by FzM1 exposure.

We reasoned that these alterations might collectively lead to a net increase in cell cycle duration, thereby slowing the kinetic progression of SCC25 cells treated with FzM1. To test this hypothesis, we performed a population kinetic analysis comparing the cell cycle length of DMSO- and FzM1-treated cells. Using thymidine analog cumulative labeling (Nowakowski et al., 1989; Martí-Clúa, 2023), cells were continuously exposed to a saturating dose of EdU, followed by staining and quantification. A short EdU pulse revealed comparable labeling indices (43.6% ± 1.6 for DMSO-treated cells and 43.4% ± 0.98 for FzM1-treated cells) indicating that a similar fraction of cells was actively cycling under both conditions (Figures 3H,I).

Over time, cumulative EdU incorporation showed that the proportion of labeled cells remained constant for approximately 10 h in DMSO-treated samples (Ts = 10h), whereas the same labeling index persisted for up to 16 h in FzM1-treated cells (Figures 3H,I). The prolonged maintenance of an unchanged EdU + fraction indicates an extended S-phase in FzM1-treated cells (Ts = 16 h). Consistently, the saturation point at which all cycling cells become EdU-positive, was also delayed following FzM1 exposure. Because this value corresponds to the total cell cycle length minus the duration of S-phase (Tc–Ts), we were able to mathematically estimate the total cell cycle duration as 31 h for DMSO-treated SCC25 cells and 42 h for FzM1-treated cells (Figures 3I,L), (Nowakowski et al., 1989; Martí-Clúa, 2023).

This significant prolongation of the cell cycle highlights a clear alteration in cell cycle regulatory mechanisms, which could substantially affect the proliferative capacity and expansion dynamics of OSCC cells.

### 3.4 FzM1 treatment alters retinoic acid metabolism and signaling to delay cell cycle progression in OSCC cells

**FIGURE 4:**
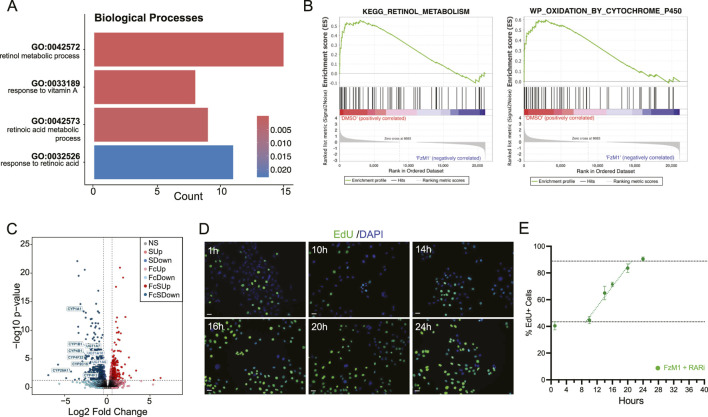
Molecular characterization of FzM1-treated cells highlights involvement of retinoic acid signaling. **(A)** GO bar diagram showing enriched biological processes in the FzM1 over DMSO condition **(B)** GSEA plots depicting significant enrichment of retinoic acid metabolism (left) and oxidation by cytochrome P450 (CYP). **(C)** Volcano plot of differentially expressed genes highlighting downregulation of multiple CYP genes and UGT family members involved in RA metabolism. NS:Not significant; SUp: Significant Up; SDown: Significant Down; FcUp: Fold Change Up; FcDown: Fold Change Down; FcSUp: Fold Change Significant Up; FcSDown: Fold Change Significant Down **(D)** Representative images of EdU-positive cells over time during cumulative incorporation assays used to calculate cell cycle progression. Scale bar: 30 µm. **(E)** Graphical representation and quantification of the percentage of EdU-positive cells over time for cell cycle analyses. Data are presented as mean ± SEM.

**FIGURE 5:**
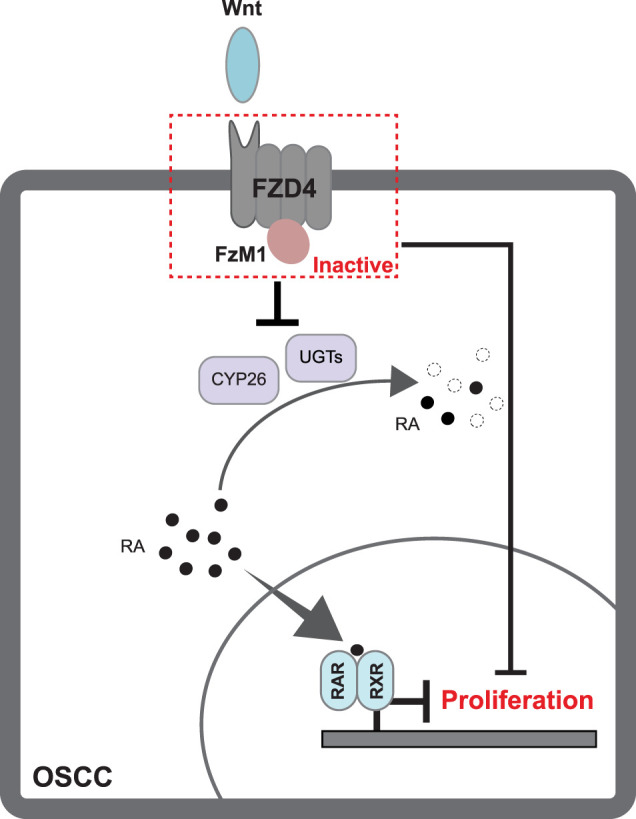
Graphical abstract. FzM1 regulates RA levels by inhibiting its oxidation and degradation, leading to RA accumulation. This stabilization of RA contributes to the control of cell cycle progression, prolonging S-phase and extending the overall cell cycle duration in an *in vitro* OSCC model.

To elucidate the molecular mechanisms by which FzM1 delays cell cycle progression in OSCC cells, we performed GO enrichment analysis of Biological Processes (BP) using our transcriptomic dataset. This analysis revealed significant enrichment of pathways related to retinoic acid (RA) metabolism and response to vitamin A (Figure 4A). Consistently, GSEA demonstrated strong enrichment of pathways associated with retinol metabolism and cytochrome P450–mediated oxidation (Figure 4B). Volcano plot analysis revealed marked downregulation of UGT and CYP genes involved in RA catabolism (Figure 4C) (Rowbotham et al., 2010; Ross and Zolfaghari, 2011), suggesting that FzM1 promotes RA accumulation by inhibiting its metabolic breakdown, thereby stabilizing intracellular RA levels (Figure 5).

Since RA is known to regulate the expression of adhesion complexes in various epithelial contexts (Aldehlawi et al., 2020; Chateau and Boehm, 1996), we investigated whether transcriptomic changes upon FzM1 treatment reflect RA-mediated alterations in the SCC25 phenotype. In line with previous reports (Miyazono et al., 2020; Kautsky et al., 1995), our data reveal a transcriptional signature marked by differential expression of genes known to be influenced by RA signaling: such as focal adhesion, adherens junctions, integrin signaling, keratin production, and collagen remodeling (Supplementary Figures S2A–C).

We next investigated the potential link between prolonged RA signaling and the delayed cell cycle progression observed following FZD4 inhibition. We reasoned that blocking RA receptor activity would reverse the effects of FzM1 and restore SCC25 cell cycle kinetics to control levels. To test this, we treated cells for 5 days with FzM1 in combination with a RA receptor antagonist (RARi). As expected, RARi completely abolished the kinetic changes observed after FzM1 treatment: Phenotypic analysis of cell-cycle dynamics revealed that FzM1+RARi–treated cells exhibit a proportion of mitotic figures comparable to DMSO controls (3.049% ± 0.45) and phospho-H3 levels restored to baseline (8.7% ± 1.69), with no statistically significance when compared to DMSO values. We then quantify cell cycle length in FzM1+RARi–treated cells. Consistently with a rescued phenotype, EdU incorporation curves revealed a S-phase duration of 10 h (Ts = 10 h) and a total cell cycle length of 31 h (Tc = 31 h), matching the values observed in DMSO-treated cells (Figures 4D,E). Through these findings, we inferred that the proliferation and cell-cycle alterations induced by FzM1 require RA pathway activation downstream of FZD4 signaling, supporting a mechanistic model in which WNT and RA pathways cooperatively regulate cell-cycle progression and OSCC cell growth.

## 4 Discussion

Dysregulated or overactive Wnt signaling is a hallmark of many human cancers, driving uncontrolled proliferation, enhanced survival, and tumor progression (Hayat et al., 2022). Given its central role in oncogenesis, the Wnt pathway represents a relevant therapeutic target, and its inhibition holds significant potential to improve treatment outcomes (Zhou et al. 2022; Zhang and Wang 2020). Several clinical trials are currently evaluating Wnt-targeted therapies, including small-molecule inhibitors and monoclonal antibodies (e.g., NCT02278133, NCT03447470), highlighting the translational relevance of this approach. Conversely, Wnt signaling is essential for adult tissue homeostasis, particularly in the maintenance of intestinal and epidermal stem cells, raising concerns about systemic toxicity associated with broad pathway inhibition (Liu et al., 2013). Targeting specific FZD receptors or other disease-relevant surface components may offer a safer, more selective alternative (Gurney et al., 2012).

FZD4, a member of the Frizzled family of G protein–coupled receptors, plays a central role in mediating Wnt signaling and has been increasingly implicated in the regulation of tumor growth and malignancy (Lin et al., 2017; Yang Y. et al., 2018; Tanaka et al., 1998; Fang et al., 2018; Yu et al., 2021). Dysregulation of FZD4 has been observed across multiple solid tumors, where it can act as an oncogenic driver (e.g., bladder, prostate, and ovarian cancers) (Chen et al., 2018; Gupta et al., 2010; Chen et al., 2018; Zhou et al., 2021) or tumor suppressor (e.g., cutaneous squamous cell carcinoma) (Zhang et al., 2021; Bian et al., 2024). Specific downregulation of FZD4 reversed prostate cancer regulating proliferation, migration and invasion (Formosa et al., 2014; Gupta et al., 2010) supporting its therapeutic potential. Neutralizing antibodies that target all FZD receptors, including FZD4, more effectively inhibit tumor growth in preclinical models than approaches that block all other FZDs while leaving FZD4 unaffected (Pavlovic et al., 2018). FzM1is a negative allosteric modulator specifically designed to inhibit FZD4 signaling, ultimately blocking the transcription of downstream T-cell factor/lymphoid enhancer-binding factor family (TCF/LEF)-dependent genes (Generoso et al., 2015).

In the present study, we provide mechanistic insight into how FzM1 modulates intracellular signaling networks and cellular behavior in OSCC. We demonstrate that FzM1 treatment results in altered expression of key cell cycle regulators and a marked delay in cell cycle progression. Quantitative cell cycle analyses revealed a significant prolongation of total cycle duration: From 31 h in OSCC cells to 42 h following FzM1 treatment, driven primarily by an extended S-phase. This kinetic slowdown reflects disrupted cycle control and reduces proliferative capacity, highlighting a direct functional consequence of FZD4 inhibition on OSCC growth dynamics. Thus, the pro-oncogenic function of FZD4 places OSCC alongside cancers such as non-small cell lung carcinoma, colon, and prostate cancer, in which FZD4 promotes tumor growth through Wnt/β-catenin signaling. In contrast, cutaneous squamous cell carcinoma represent a notable difference, where FZD4 overexpression inhibits proliferation and induces apoptosis. These observations underscore the context-dependent nature of FZD4 signaling, highlighting its oncogenic activity in OSCC and other epithelial malignancies.

Mechanistically, our transcriptomic profiling revealed a strong association between FZD4 inhibition and the RA metabolic pathway. FzM1-treated cells exhibited downregulation of cytochrome P450 (CYP) enzymes and glucuronosyltransferases (UGTs), both key mediators of RA catabolism. This suppression likely elevates intracellular RA levels by stabilization, potentially linking the FzM1-RA axis to the observed deceleration of the cell cycle (Figure 5). Previous studies have shown that Wnt activity can induce CYP26A1 expression, reducing RA signaling, impairing cellular differentiation, and promoting continued stem cell proliferation, thereby contributing to tumor development (Liu et al., 2002; Easwaran et al., 1999; Chanda et al., 2013). Additionally, UGTs are known to participate in the metabolism of diverse classes of cancer therapeutics (Allain et al., 2020), suggesting that their regulation by FzM1 could have broader implications for cancer therapy. Together, these findings further support a role for Wnt–FZD4 signaling within the broader regulatory framework of OSCC.

Elevated RA availability is known to affect key regulators of cell cycle progression, such as cyclin turnover, MAPK signaling, and Rb phosphorylation (Crowe et al., 2003). Notably in our study, co-treatment of FzM1 with the inhibitor of retinoic acid receptors AGN 193109 (RARi) largely restored basal cell cycle kinetics, reversing the delay observed with FzM1 alone. This effect indicates that FzM1-induced slowing of the cell cycle depends on RA receptor activation, supporting a model in which RARi acts downstream of Wnt-FZD4 signaling axis.

Similar to our OSCC model, RA shows promising anti-cancer activity in multiple contexts. In colitis-associated colorectal cancer, RA levels are markedly reduced due to altered metabolism, with inhibition of RA signaling promoting tumorigenesis. Notably, elevated expression of the RA-catabolizing enzyme CYP26A1 correlated with poorer prognosis in patients (Bhattacharya et al., 2016). In lung squamous cell carcinoma, RA treatment of human cancer cells and tumor xenografts suppresses proliferation while promoting apoptosis and differentiation (Moro et al., 2015). Despite these preclinical results, retinoids have not achieved broad clinical utility in patients; however, novel combinatorial strategies and modern formulations may offer opportunities to improve efficacy (Tripathi et al., 2019).

In summary, our findings provide a comprehensive view of the multifaceted mechanisms through which FzM1 exerts its antitumor effects in OSCC. By selectively inhibiting FZD4, FzM1 simultaneously disrupts Wnt-dependent oncogenic signaling, alters RA metabolism and impairs regulators of cell cycle progression. These converging effects culminate in a substantial prolongation of the cell cycle length ultimately suppressing cell proliferation. Future work to confirm these findings is planned, using patient-derived organoids and *in vivo* studies to validate and extend the impact of the *in vitro* results. Collectively, our findings position FzM1 as a promising therapeutic strategy for FZD4-overexpressing malignancies and highlight the potential of targeting the FZD4-RA axis in oral squamous cell carcinoma.

## Data Availability

The datasets presented in this study can be found in online repositories. The names of the repository/repositories and accession number(s) can be found below: https://www.ncbi.nlm.nih.gov/genbank/, PRJNA1355448.

## Glossary

Wnt: Wingless and Int-1
FZD4: Frizzled 4
OSCC: Oral squamous cell carcinoma
HPV: human papillomavirus
EGFR: epidermal growth factor receptor
MAPK: mitogen-activated protein kinase
NF-κB: Nuclear Factor kappa-light-chain-enhancer of activated B cells
Bcl-2: B-cell lymphoma 2
Rb: retinoblastoma
CDK: cyclin-dependent kinase
TP53: Tumor Protein p53
E2F: Early 2 Factor
miRNA: microRNAs
lncRNA: long non-coding RNAs
circRNA: circular RNAs
LRP5/6: low-density lipoprotein receptor-related proteins 5 and 6
EMT: epithelial-to-mesenchymal transition
RA: retinoic acid
4NQO: 4-nitroquinoline 1-oxide
DMSO: Dimethyl sulfoxide
PFA: paraformaldehyde
PBS: Phosphate-buffered saline
FBS: fetal bovine serum
DAPI: 4′,6-diamidino-2-phenylindole dihydrochloride
ATCC: American Type Culture Collection
DMEM/F12: Dulbecco’s Modified Eagle Medium Nutrient Mixture F-12
HEPES: 4-(2-hydroxyethyl)-1-piperazineethanesulfonic acid
EDTA: Ethylenediaminetetraacetic acid
FzM1: 3-Hydroxy-5-[[(2-Naphthalenylamino)carbonyl]amino]benzoic acid
RARi: Retinoic acid receptor inhibitor
pH3: phospho Histone3
EdU: 5-Ethynyl-2′-deoxyuridine
Tc: Total cell cycle length
Ts: S-phase length
RIPA: Radioimmunoprecipitation assay
DTT: dithiothreitol
SDS-PAGE: Sodium Dodecyl Sulfate-PolyAcrylamide Gel Electrophoresis
TBS: Tris-Buffered Saline
BSA: Bovine Serum Albumin
GAPDH: glyceraldehyde-3-phosphate dehydrogenase
HRP: Horseradish Peroxidase
Krt10: Keratin10
TBS-T: Tris-Buffered Saline- Tween 20
KEA: Kinase Enrichment Analysis
GO: Gene Ontology
GSEA: Gene set enrichment analysis
CDK1: Cyclin-dependent kinase 1
CDK2: Cyclin-dependent kinase 2
BP: Biological Processes
TCF/LEF: T-cell factor/lymphoid enhancer-binding factor family
CYP450: cytochrome P450
UGTs: glucuronosyltransferases
DEG: differentially expressed genes
ORA: over-representation analysis
SEM: Standard Error of the Mean
WP: WikiPathways
KEGG: Kyoto Encyclopedia of Genes and Genomes
RIN: RNA Integrity Number
FPKM: fragments per kilobase of transcript per million mapped reads
DEG: Differentially Expressed Genes
