# Supplementary figures and images for: Pharmacological inhibition of frizzled 4 delays cell cycle progression and limits oral squamous cell carcinoma growth
Source: Front Cell Dev Biol. 2026 Feb 17;14:1756565. doi: 10.3389/fcell.2026.1756565 (PMC12953554; doi:10.3389/fcell.2026.1756565)

Supplementary Figure 2

A

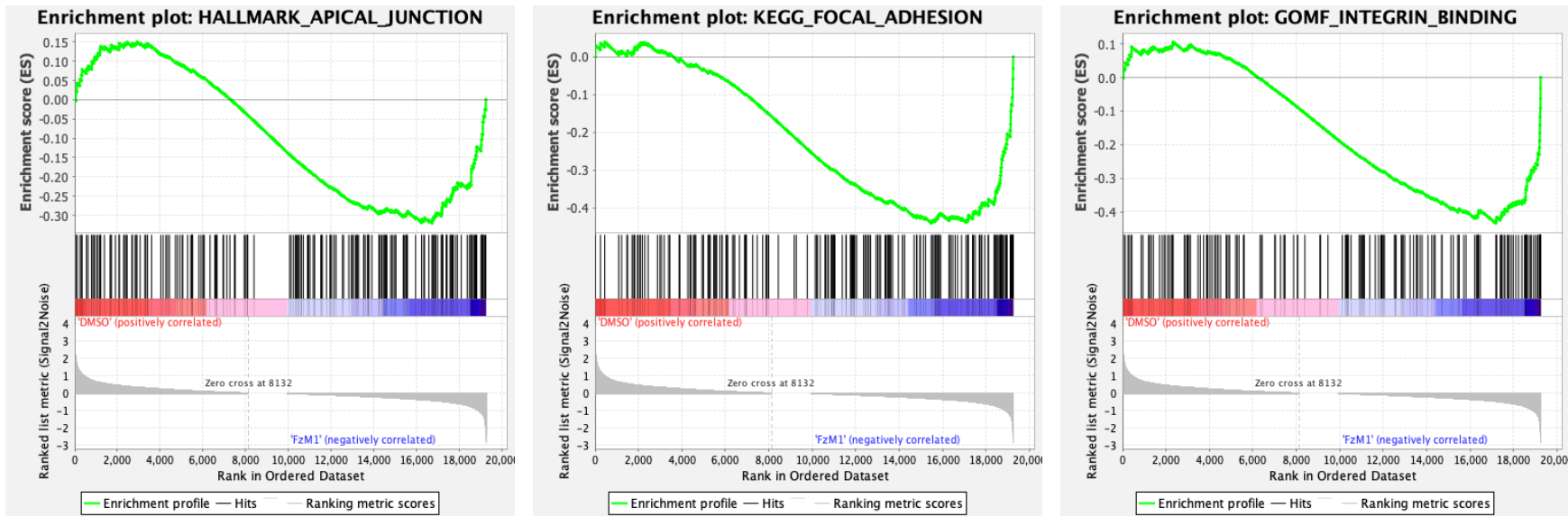

B

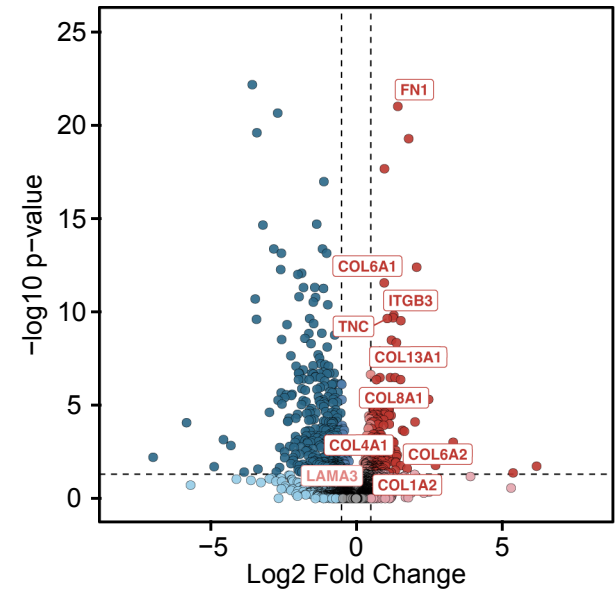

C

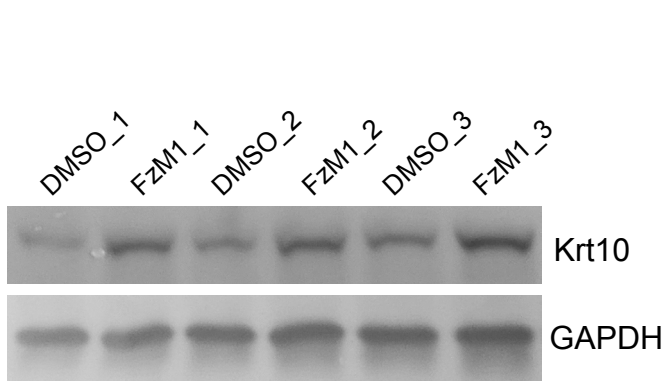

Supplement: Supplementary file 1 [file DataSheet2.pdf]

Supplementary Figure 1

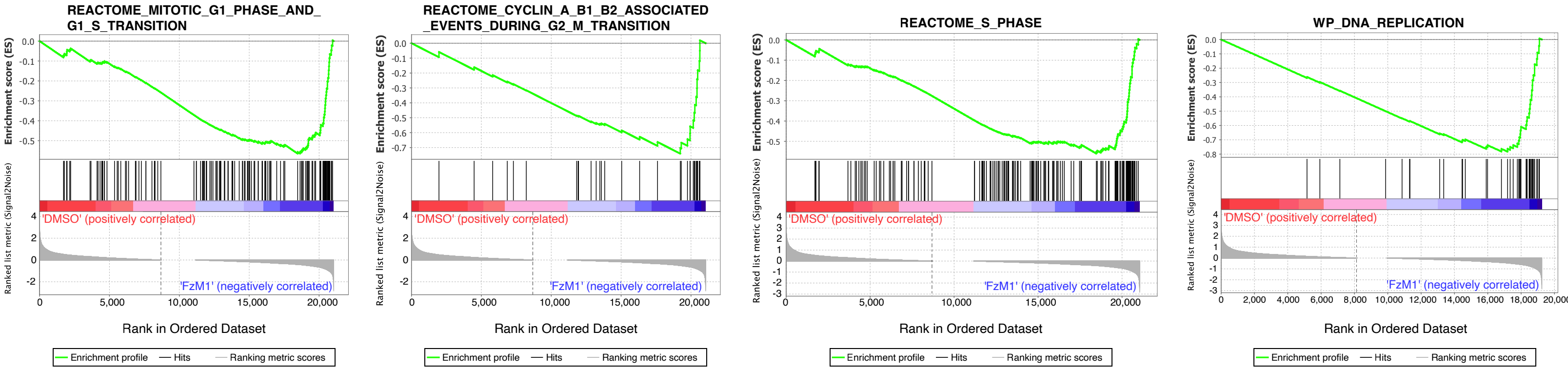

Supplement: Supplementary file 5 [file DataSheet1.pdf]
