# Supplementary material for: Pharmacological inhibition of frizzled 4 delays cell cycle progression and limits oral squamous cell carcinoma growth
Source: Front Cell Dev Biol. 2026 Feb 17;14:1756565. doi: 10.3389/fcell.2026.1756565 (PMC12953554; doi:10.3389/fcell.2026.1756565)
Supplement: Supplementary file 3 [file Table1.docx]

**Supplementary Table 1: Human primer pairs for quantitative real-time PCR**

| **Target** | **FW primer 5’-3’** | **RV primer 3’-5’** |
| --- | --- | --- |
| hGADPH | gcatcctgggctacactgag | ccaccaccctgttgctgtag |
| hActin | gcactcttccagccttcctt | ctccttctgcatcctgtcgg |
| hFZD4 | cgtgaccaagatgcccaacc | acgggttcacagcgtctctt |
| hCDK1 | ctacaggtcaagtggtagcca | gaagaatccatgtactgaccagg |
| hCDK2 | gctgtggacatctggagcct | tgcgataacaagctccgtcca |
| hCCNA1 | cggcacactcaagtcagacc | tccgtgatgtctggctgctt |
| hCCNA2 | agcactctacacagtcacggg | agtgtctctggtgggttgagg |
| hCCNE1 | ccaaaatcgacaggacggcg | ggagcctctggatggtgcaa |
| hCCNE2 | ccccaagaagcccagataatcca | ccccagcttaaatcaggcaaagg |
| hCCND2 | gctcgctcacttgtgatgcc | caggtcgatatcccgcacgt |
