# Supplementary material for: Pharmacological inhibition of frizzled 4 delays cell cycle progression and limits oral squamous cell carcinoma growth
Source: Front Cell Dev Biol. 2026 Feb 17;14:1756565. doi: 10.3389/fcell.2026.1756565 (PMC12953554; doi:10.3389/fcell.2026.1756565)

Supplementary Method

A

Between-group comparison

	Number
P-value threshold:	p <= 0.01
Log ratio threshold:	log ratio >= 0.5
Number of significant genes:	834

Number of significants by p-value and fold-change

	#significants	FDR	fc >= 1	fc >= 1.5	fc >= 2	fc >= 3	fc >= 4	fc >= 8	fc >= 10
p < 0.1	2615	0.5520000	2615	1227	512	170	81	21	19
p < 0.05	1967	0.3669000	1967	1044	452	163	78	21	19
p < 0.01	1127	0.1281000	1127	725	327	134	70	18	17
p < 0.001	605	0.0237800	605	465	224	95	51	12	11
p < 1e-04	373	0.0037250	373	315	169	76	39	9	8
p < 1e-05	242	0.0005818	242	217	132	58	31	6	5

B

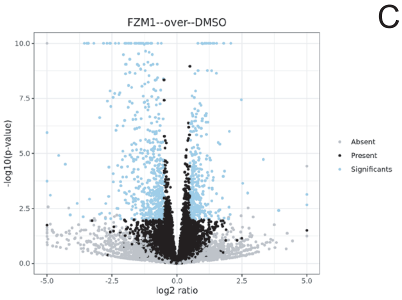

C

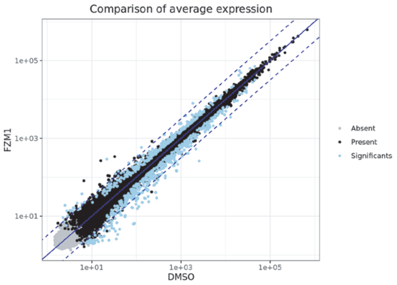

Supplement: Supplementary file 4 [file DataSheet3.pdf]
